# Pharmacokinetic and pharmacodynamic modeling of oral mitiglinide on glucose lowering in healthy Chinese volunteers

**DOI:** 10.1186/s40360-017-0161-6

**Published:** 2017-07-04

**Authors:** Shijia Liu, Peidong Chen, Yang Zhao, Guoliang Dai, Bingting Sun, Yao Wang, Anwei Ding, Wenzheng Ju

**Affiliations:** 1 Affiliated Hospital of Nanjing University of Chinese Medicine, Nanjing, Jiangsu 210029 China; 2 Nanjing University of Chinese Medicine, Nanjing, Jiangsu 210016 China; 3 Office of Pharmaceutical Quality, Center of Drug Evaluation and Research, U.S. Food and Drug Administration, 10903 New Hampshire Avenue, Silver Spring, MD 20903 USA

**Keywords:** Mitiglinide, Pharmacodynamic, Pharmacokinetic, Pharmacokinetic–pharmacodynamics modeling, Plasma glucose

## Abstract

**Background:**

Mitiglinide is a widely used agent for diabetic treatment. We established a pharmacokinetic-pharmacodynamic (PK-PD) model to illustrate the relationship between mitiglinide plasma concentration and its glucose lowering effects in healthy volunteers.

**Methods:**

The volunteers participated in the test after the administration of a single dose of 10 mg mitiglinide. The drug concentration in Plasma and the values of glucose levels were determined by LC-MS/MS assay and hexokinase method. A PK-PD model was established with a series of equations to describe the relationship between plasma medicine and glucose, and the equations were solved numerically and fitted to the data with the Phoenix NLME software.

**Results:**

The results of the two-compartment model analysis were based on the maximum likelihood criterion and visual inspection of the fittings. The terminal elimination half-life (*t*_1/2_) was 1.69 ± 0.16 h and the CL/F was 7.80 ± 1.84 L/h. The plasma glucose levels began to decline by 0.2 h, and hit its bottom decreasing values of 2.6 mg/L at 0.5 h after administration. The calculated parameter and fitting curve indicated that the model established in our experiment fitted well.

**Conclusions:**

A PK/PD model illustrates that the relationship between mitiglinide concentration in plasma and glucose lowering effect in healthy volunteers was established. The results of our experiment suggested that the model can be used reasonably to predict the relationship between PK and PD in mitiglinide, which could be used in diabetes mellitus dosage control in clinical trials and other fields.

## Background

Type 2 diabetes mellitus, which affects approximately 11% of adults in the U.S. and 8% worldwide [1], is resulted from a progressive decline in insulin action and the reduced response of β-cells to insulin [2, 3]. ATP-sensitive K^+^ (K_ATP_) channels exists widely in pancreatic cells, cardiac, smooth and skeletal muscles, and central nervous system [4]. Different K_ATP_-channel may exist in different tissues, Kir6.2/SUR1 for β-cells, some neurons cardiac, Kir6.2/SUR2A for skeletal K_ATP_-channels, and Kir6.2/SUR2B for vascular smooth muscle K_ATP_-channels [5].

Mitiglinide is a rapid acting drug for diabetic drugs, which is selectively active on K_ATP_ channel of Kir6.2/SUR1 [6, 7]. Previous study has demonstrated that the preprandial administration of mitiglinide was benefit for postprandial hyperglycemia and glycemic control [8]. A series of sensitive HPLC-MS/MS methods have been used to determine the pharmacokinetics (PK) properties of mitiglinide in rats [9] and humans. The PK property of 24 volunteers who received oral administration mitiglinide at a single dose from 5 to 20 mg/kg was studied [10]. The maximum plasma concentration (*C*_*max*_) in volunteers with mitiglinide from 5 to 20 mg/kg was 565.30 and 2625.26 ng/mL respectively, and the terminal half-life (*T*_*1/2*_) was 1.19 and 1.43 h respectively. Pharmacokinetic–pharmacodynamic population model-based studies may suggest an accurate model to provide information for impact assessment of the treatment [11]. The relationship between the pharmacokinetics of mitiglinide and the drug effects has not been studied in humans. The objective of present study is to measure the relationship between plasma drug concentration and its effects on healthy volunteers with oral administration. The experiment result may be used for prediction the therapeutic active of mitiglinide and its side effect profiles in clinic.

## Methods

### Drug

Mitiglinide (10 mg/tablet, lot no.14032011, expiration: 29/02/2016) was manufactured by Suzhong Pharma Co. Ltd., Jiangsu, China.

### Subjects

The study was approved by the Ethics Committee of the Affiliated Hospital of Nanjing University of Chinese Medicine (IRB#2014NL-020-02). Eighteen healthy male volunteers participated in this study, with average age of 24 (ranging from 18 to 40) and average weight of 67.5 kg (ranging from 56 to 80 kg). All volunteers went through thorough physical examinations and routine laboratory evaluations, including haematology, serum chemistry, virus and urinalysis. None of the volunteers in this study took any drugs which would have affected this study for at least 3 months beforehand. The limitation of our study lies in the fact that subjects were excluded from health problems including drug or alcohol abuse, and the abnormalities in the experiment. All volunteers were informed of the purpose and possible risks before the test and they could withdraw from the test any time for any reason. Signed informed consent was obtained before any trial-related activities.

### Experimental design

Subjects were fasted for 10 h before the experiment. An intravenous cannula was inserted into the forearm vein to collect blood samples at time zero. The test group took the agent with 250 mL of water after baseline sampling. All subjects were accommodated in the care unit in the Affiliated Hospital of Nanjing University of Chinese Medicine during the test. Blood samples were taken at 0, 0.08, 0.17, 0.25, 0.33, 0.5, 0.75, 1, 1.5, 2, 3 h after drug administration, centrifuged immediately for 10 min at 3000 rpm, and stored at −80 °C until analysis.

### Analysis of the plasma drug concentrations and blood glucose levels

Plasma mitiglinide concentration was determined by the modification of the methods of Yu and Takanohashi [9, 12]. A 1200 HPLC system (Agilent technologies, Palo Alto, CA, USA) coupled with a triple-quadrupole tandem API 4000 mass spectrometer (AB/MDS-Sciex, Concord, Ontario, Canada) were used for analysis. Instrumental control and data processing was performed by the Analyst software (version 1.4.2). The LC separation was performed on an Agilent Zorbax SB-C_18_column (150 mm × 2.1 mm I.D., 3.5 μm, Agilent Technologies, Wilmington, DE, USA) with a security guard column (12.5 mm × 2.1 mm I.D., 5 μm, Agilent Zorbax SB-C_18_, DE, USA). The mobile phase consisted of methanol and deionized water (*v:v*, 60:40) containing 0.1% formic acid at a flow rate of 0.30 mL/min, the autosampler temperature was set at 15 °C and the column temperature was set at 30 °C. A MS detector with electrospray ionization (ESI) interface was used in our experiment and the positive ion mode was selected for quantitative analysis. Quantitation was detected by the multi-reaction-monitoring (MRM) mode of transitions of m/z 316.2 → 298.2 for Mitiglinide, m/z 318.2 → 120.2 for Nateglinide. The optimized conditions used for the ESI^+^ source were set as follows: capillary voltage 5.5 KV; turbo heater temperature 600 °C; curtain gas (CUR) 40 psi; collision activation dissociation (CAD) 8 psi; declustering potential (DP) 81 V; collision energy (CE) 23 eV for mitiglinide and 29 eV for IS respectively. Blood glucose concentration was examined by the glucose oxidase method with the use of an autoanalyzer (TosohG8, Japan).

### Statistical analysis

Graphical and all other statistical analyses, including the evaluation outputs, were performed within the Phoenix platform [13].

### Pharmacokinetic/pharmacodynamic model and data analysis

**Fig. 1:**
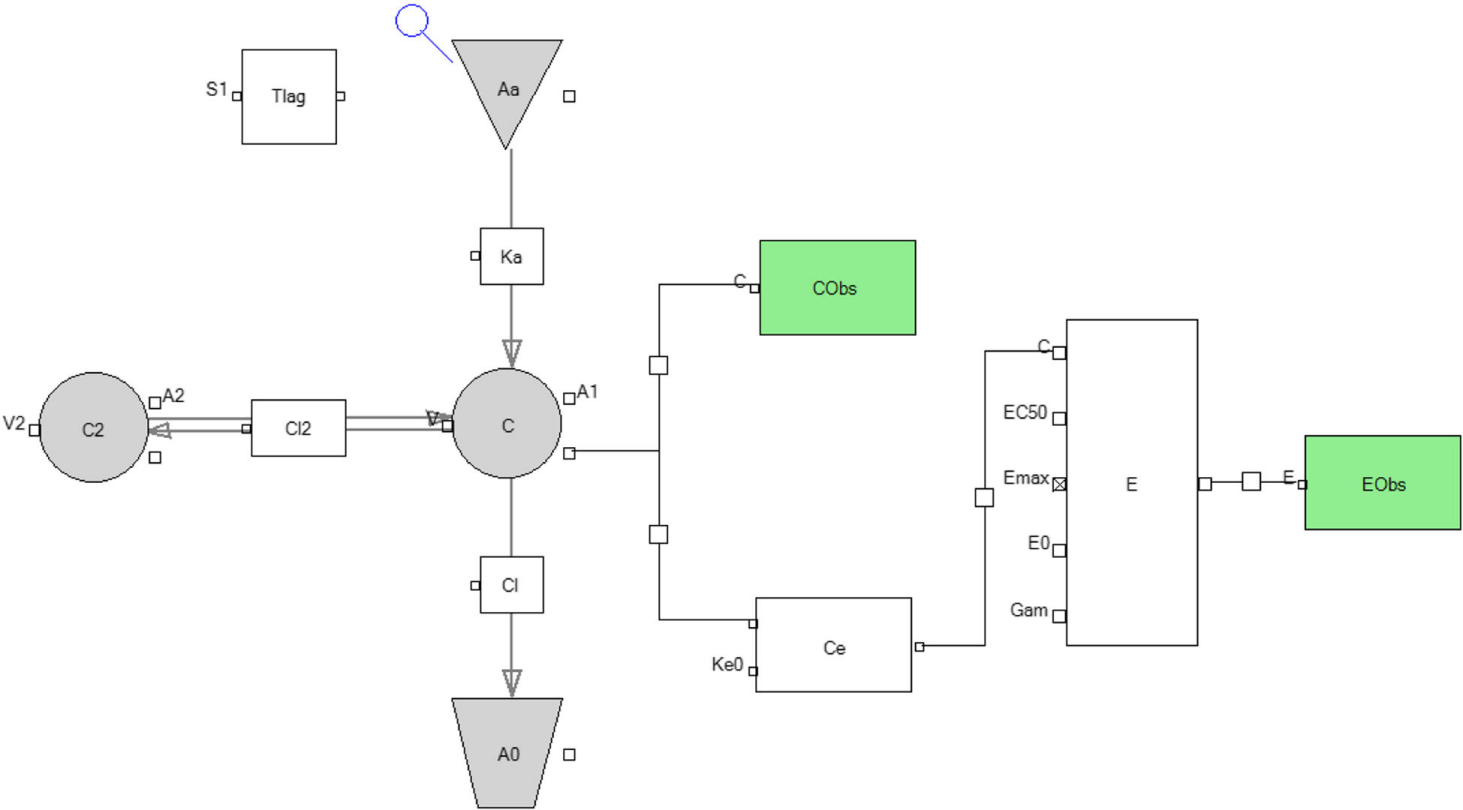
Scheme of the pharmacokinetic-pharmacodynamic (PK–PD) model. A1, mitiglinide amounts in the central compartment; A2, mitiglinide amounts in the peripheral compartment; C, mitiglinide concentration in the central compartment; C2, mitiglinide concentration in the peripheral compartment; V, central volume of the distribution of mitiglinide; V2, peripheral compartment; ke0, glucose disappearance rate constant; E, level of glucose in the plasma, E0, blood glucose maintained at a certain level in absence of the drug; Ce, plasma concentration in the effect compartment

A two-compartment linked with E_max_ PK-PD model was implemented by maximizing the log-likelihood using the first-order conditional estimation (FOCE) method of the Phoenix NLME software (Pharsight Corp, St. Louis, Missouri, United States of America). Pharmacokinetics and pharmacodynamics values of mitiglinide were modelled sequentially. The models were established as a series of equations, describing the relationship between plasma medicine and glucose. The equations were solved numerically and fitted to the data with Phoenix NLME [11]. Plasma concentration of mitiglinide was used for the PK analysis. The two-compartment model for oral administration was established by the least-squares method, and the pharmacokinetic parameters including V, V2, CL and CL_2_ were also calculated. The amounts of mitiglinide and plasma concentrations in the central compartment (C) were determined and calculated by the distribution volume for the central compartment (V). The two-compartment model as shown in Fig. 1, and the overall PK model could be described as Eqs. (1) to (11) as below:

1 $$ \mathrm{dAa}/\mathrm{dt}=\hbox{-} {\mathrm{Ka}}^{\ast }\ \mathrm{Aa} $$

2 $$ \mathrm{dA}1/\mathrm{dt}={\mathrm{Ka}}^{\ast }\ \mathrm{Aa}\hbox{-} {\mathrm{CL}}^{\ast }\ \mathrm{C}\hbox{-} \mathrm{C}\mathrm{L}{2}^{\ast }\ \left(\mathrm{C}\hbox{-} \mathrm{C}2\right) $$

3 $$ \mathrm{dA}2/\mathrm{dt}=\mathrm{CL}{2}^{\ast }\ \left(\mathrm{C}\hbox{-} \mathrm{C}2\right) $$

4 $$ \mathrm{C}=\mathrm{A}1/\mathrm{V} $$

5 $$ \mathrm{C}2=\mathrm{A}2/\mathrm{V}2 $$

6 $$ \mathrm{Ka}={\mathrm{tvKa}}^{\ast }\  \exp \left(\upeta \mathrm{Ka}\right) $$

7 $$ \mathrm{V}={\mathrm{tvV}}^{\ast }\  \exp \left(\upeta \mathrm{V}\right) $$

8 $$ \mathrm{V}2=\mathrm{tvV}2 $$

9 $$ \mathrm{CL}=\mathrm{tvCL} $$

10 $$ \mathrm{CL}2=\mathrm{tvCL}{2}^{\ast }\  \exp \left(\upeta \mathrm{CL}2\right) $$

11 $$ \mathrm{Tlag}={\mathrm{tvTlag}}^{\ast }\  \exp \left(\upeta \mathrm{Tlag}\right) $$

A means the amount of mitiglinide, A_1_ and A_2_ suggest the amounts of drug in the central and peripheral compartments respectively. The concentrations of mitiglinide in the central and peripheral compartment are represented as C and C_2_ respectively. The elimination clearances of the drug in the central and peripheral compartments are represented as CL and CL_2_ respectively. TV indicates the typical value of population mean, and η indicates the inter-individual variation. PK parameters were obtained by simultaneously fitting the plasma concentration data after oral administration of mitiglinide (10 mg) to volunteers using the 2-compartment PK model (Table 1).

An Emax PD model was selected based on previous research on describing the relationship between mitiglinide concentrations and the glucose levels in the plasma [14–16]. The Biophase model which consists of compartmental PK model in conjunction with Emax PD model is one of the commonly used PK-PD models. If biophase model could adequately fit both the PK and PD profiles, it would render it possible for further application. The glucose plasma concentration (mg/mL) was the response variables in the experiment, and the model can be described by equations as shown below:

12 $$ \mathrm{dCe}/\mathrm{dt}=\mathrm{Ke}{0}^{\ast}\left(\mathrm{C}\hbox{-} \mathrm{Ce}\right) $$

13 $$ \mathrm{E}=\mathrm{E}{0}^{\ast}\;\left(1\hbox{-} {\mathrm{Ce}}^{\wedge}\mathrm{Gam}/\left(\mathrm{IC}{50}^{\wedge}\mathrm{Gam}+{\mathrm{Ce}}^{\wedge}\mathrm{Gam}\right)\right) $$

14 $$ \mathrm{Ke}0=\mathrm{tvKe}{0}^{\ast }\  \exp \left(\upeta \mathrm{Ke}0\right) $$

15 $$ \mathrm{IC}50=\mathrm{tvIC}50 $$

16 $$ \mathrm{Gam}={\mathrm{tvGam}}^{\ast }\  \exp \left(\upeta \mathrm{Gam}\right) $$

17 $$ \mathrm{E}0=\mathrm{tvE}{0}^{\ast }\  \exp \left(\upeta \mathrm{E}0\right) $$

Where E is the level of glucose in the plasma, E_0_ is the blood glucose maintained at a certain level in absence of the drug. C is the plasma concentration in the central compartment and Ce represents the plasma concentration in the effect compartment. K_eo_ represents the glucose disappearance rate constant. TV represents the typical value of the population mean and η means the inter-individual variation, and the inter-individual variation for parameters with high shrinkage value (>0.5) were not included in the model.

## Results

**Fig. 2:**
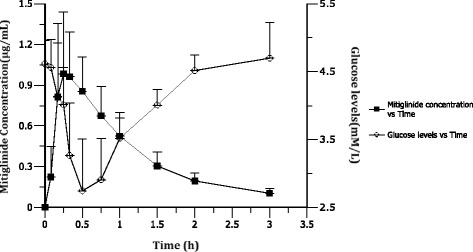
Plasma mitiglinide concentration and plasma glucose concentration after a single oral dose of 10 mg mitiglinide to healthy volunteers (mean ± SD, *n* = 18)

The plasma concentration vs time curve following oral administration of 10 mg of mitiglinide was shown in Fig. 2. Solid line represents the mean concentration of the test coupled with the standard deviation on each time point. Terminal elimination half-life (*t*_1/2_) is 1.69 ± 0.16 h and the CL/F 7.80 ± 1.84 L/h. Figure 2 also shows the glucose profiles after drug administration, the model independent parameters are calculated by using individual approach with Non compartmental analysis, and the model dependent parameters are obtained by using population approach with clearance parameterization. The plasma glucose levels declined after a lag of 0.2 h, and hit its bottom values of 2.6 mg/L by 0.5 h after administration. Mitiglinide declined from 0.2 to 2.2 h after administration. PK parameters were obtained by simultaneously fitting the plasma concentration data after oral administration of mitiglinide to volunteers using the 2-compartment model.

**Fig. 3:**
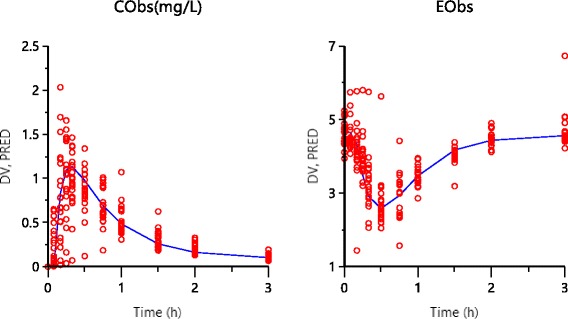
Population fitting of plasma mitiglinide concentration and plasma glucose concentration after a single oral dose of 10 mg mitiglinide to healthy volunteers (Left: Population prediction of plasma concentration vs time, Right: Population Prediction of glucose concentration vs time). The solid line is the best fit of the PK-PD model. (DV, Dependent value; PRED, Population prediction)

The two-compartment model analyzes the results based on the maximum likelihood criterion and visual inspection of the fittings. The data was calculated by the first-order absorption with a lag time and linear elimination. Non-compartmental and population modeling parameters are listed in Tables 1 and 2. PK/PD was analyzed based on the pharmacokinetic parameters obtained from the test as an input function. Figure 3 illustrates the fitting result of the plasma concentration and plasma glucose levels with population PK-PD model where the solid line represents the best fit of the model.

**Table 1:** Non-compartmental parameters for mitiglinide after a single 10 mg oral dose in healthy volunteers (mean ± SD, *n* = 18)

Parameter	Mean ± SD
*t*_1/2_ (h)	1.69 ± 0.16
*T*_max_ (h)	0.25 (0.17–0.50)
CL/F (L/h)	7.80 ± 1.84
Vd/F (L)	23.96 ± 7.23
*C*_max_(ng/mL)	1165.10 ± 357.50
AUC _0-t_ (ng/mL·h)	1489.60 ± 336.85
AUC_0-∞_ (ng/mL·h)	1497.20 ± 339.73
AUC_0-t_ / AUC_0-∞_ (%)	99.51 ± 0. 16

**Table 2:** Population modeling parameters for mitiglinide after a single 10 mg oral dose to healthy volunteers (mean ± SD, *n* = 18)

Parameter	Mean ± SD	Inter-individual variability (CV %)
Ka (/h)	9.57 ± 2.38	91.95
Tlag (h)	0.09 ± 0.01	70.71
V (L)	6.15 ± 0.33	17.61
CL (L/h)	0.03 ± 0.01	-
V2 (L)	104.69 ± 11.37	-
Cl2 (L/h)	9.85 ± 0.51	22.18
IC50 (μg/mL)	1.13 ± 0.08	-
E0 (mmol/L)	4.65 ± 0.09	4.35
Ke0 (/h)	7.47 ± 1.89	90.61
Gamma	1.66 ± 0.18	18.67

**Fig. 4:**
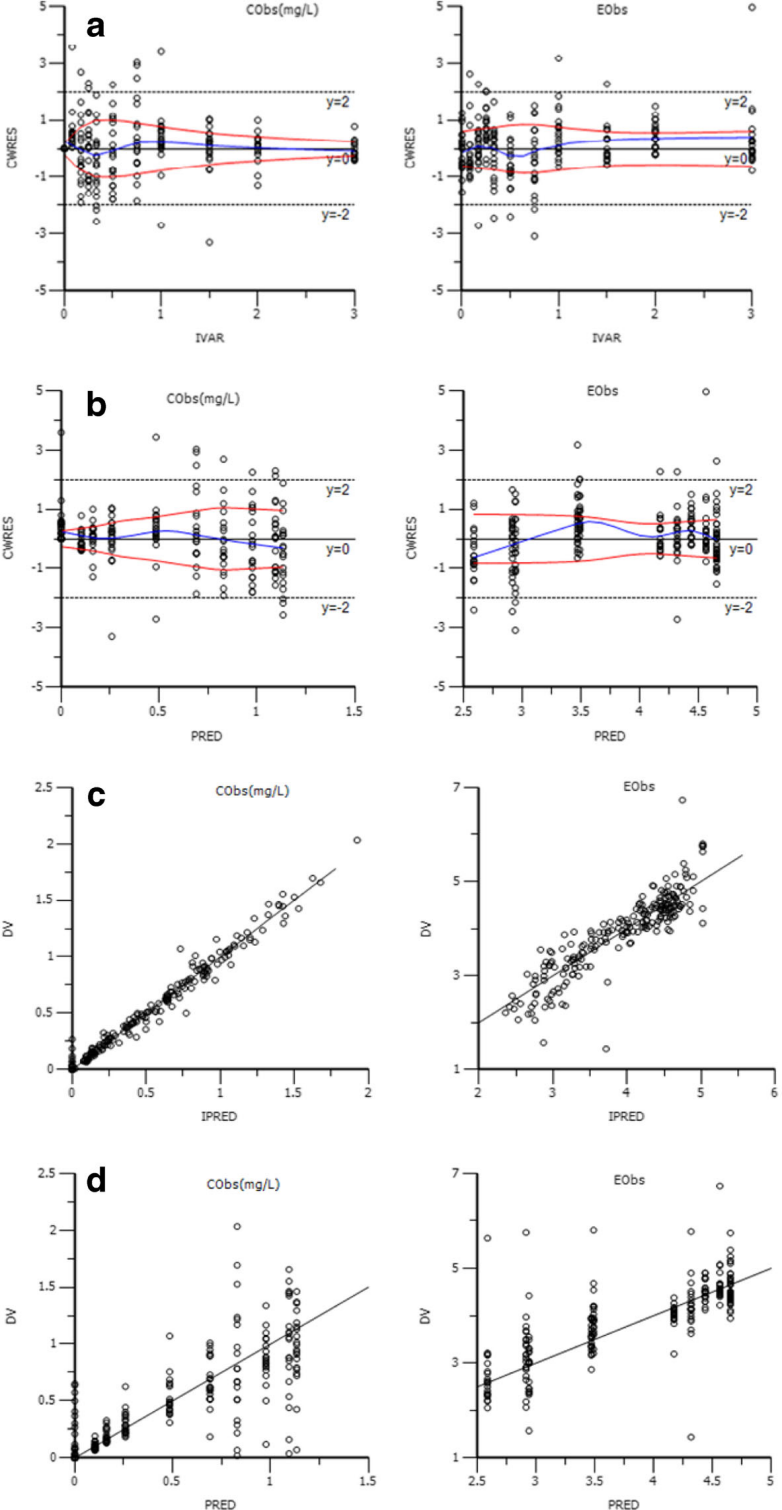
Diagnostic plots of the final Population PK-PD model for mitiglinide from studies. Diagnostic plots include (**a**) Conditional weighted residuals (CWRES) and independent variable (IVAR), (**b**) weighted residuals versus predicted values (PRED), (**c**) Plot of the observed value (DV) versus individual population predicted values (IPRE) and (**d**) The observed value versus population predicted values (PRED)

The diagnosis chart of final model was shown in Fig. 4. A is the scatter plots which represents the conditional weighted residuals (CWRES) and independent variable (IVAR). The CWRES distribution of the final model is narrow, and uniform on both sides of the zero line, which indicates that the model fitted well. B is the final model CWRES on the PRED of the scatter diagram. The value of CWRES ranged between 2 and −2 indicating the minor differences in prediction model. C is the observed value (DV) of individual predicted value (IPRE) scatter diagram and D is the observed concentrations (DV) versus population predicted values (PRED).

Based the results above, the two-compartment PK model of first-order absorption with a lag time and linear elimination conjunction with biophase PD model was developed which could capture both PK and PD profiles of mitiglinide in the present reasonably well.

## Discussion

### Pharmacokinetics

Mitiglinide could reduce the early-phase insulin release and promote the controlling of the postprandial glucose levels with its ability to fasten insulin secretagogue [17]. The characteristic of mitiglinide of anti-hyperglycemic can take effect more rapidly compared with sulphonylureas. The fast release of insulin within a few minutes was due to the rapid absorption rate of mitiglinide after oral administration [18]. In the 90% confidence internal of geometric mean ratios, mitiglinide reached its peak plasma concentration (*C*_max_) and AUC_last_, which represented the area under the plasma concentration-time curve from time zero to the time of the last measurable concentration, were observed as 0.9694 (0.8120, 1.1573) and 0.8951 (0.8440, 0.9494), respectively [19]. Mitiglinide could bind to the SUR and activate it in the pancreatic β-cells, which was considered as the mechanism of the drug underlying the therapeutic effect [20, 21]. In the present study, mitiglinide was given by oral administration and the short elimination half-life, *t*_1/2_ (h) was 1.69. Therefore, the fast absorption in the first 2 h may be caused by the binding of the free form drug to the SUR. The total clearance (CL/F) was 7.79 mL/h in our study, which is comparable to rat (0.83 mL/h) in previous studies [22].

### PK-PD modeling of glucose lowering effects

The characters of PK behaviors and its influences to the values of plasma glucose levels have been studied in several hypoglycemic drugs [23, 24]. Toshiyuki Takanohashi et al. reported the pk-pd. modeling methodology to describe the rapidity therapeutic effect of mitiglinide by using the receptor-binding-dissociation in rats [12]. However, model of inhaled glucose in healthy humans has not been published previously. Phoenix platform is the commonly used software to investigate PK/PD characters depending on the 2-compartment model [25, 26]. Our study analyzed the effects of mitiglinide on glucose lowering after a single oral dose. The shorter duration action of mitiglinide and the decreasing glucose levels in plasma was described by a two-compartment model followed by the least-squares method. The fitting results were adequate, and the efficacy of the drug has been appropriated proposed by the empirical biexponential equation. Furthermore, the model is flexible for the concentration and efficiency of the drug. The *T*_*max*_ (h) of mitiglinide was 0.25 (0.17–0.50) in our study and the glucose decrease reached its maximum.

Although mitiglinide is a commonly used and effective drug for diabetes mellitus in clinic, its side effects and the interaction remain to be studied further. A recent investigation shows that mitiglinide treatment could significantly decrease plasma fibroblast growth factor levels, which would have various effects on pancreatic islets [27]. Our results may illustrate the physiological effects of mitiglinide and its relationship with plasma concentration. Different kinds of indirect response models can be used to explain the pk-pd. property of drug. In our study, we investigated glucose disappearance and mitiglinide concentration levels in plasma by building the pk-pd. modeling. The CWRES distribution of the final model uniformed on both sides of the zero line, and the value ranged between 2 and −2 indicating the minor differences in prediction model. The result of our research could describe the drug’s pharmacological action. The results of our experiment are benefit for study designs and appropriate dosage selection of diabetes mellitus in clinical trials.

## Conclusions

A PK/PD model illustrates that the relationship between mitiglinide concentration in plasma and glucose lowering effect in healthy Chinese volunteers was established. The PK data of mitiglinide after oral administration were analyzed by a two-compartment model of first-order absorption with a lag time and linear elimination. The results of our experiment suggested that the model can be used reasonably to predict the relationship between PK and PD in mitiglinide. The established PK-PD model may be used to describe the drug’s pharmacological action. The results of our experiment are flexible for the concentration and efficiency of the drug and may be used for study designs and diabetes mellitus dosage control in clinical trials.
